# Combined Mechanisms of *Streptomyces* sp. HU2014 and Coronatine in Promoting Maize Seedling

**DOI:** 10.3390/microorganisms14061361

**Published:** 2026-06-17

**Authors:** Linfeng Hu, Xiaoyu Wang, Jiangsheng Meng, Qian Su, Wenhui Shi, Jungao Zhang, Hongxia Zhu

**Affiliations:** 1 Institute of Agricultural Resources and Environment, Academy of Agricultural Sciences of Xinjiang Uygur Autonomous Region, Urumqi 830091, China; hulinfeng@xaas.ac.cn (L.H.); 17881273601@163.com (X.W.); 18393873586@163.com (Q.S.); xjylswh@163.com (W.S.); 2 Xinjiang Crop Chemical Control Engineering Technology Research Center, Urumqi 830091, China

**Keywords:** maize, *Streptomyces* sp. HU2014, coronatine, plant growth promotion, jasmonic acid

## Abstract

The rhizosphere microbiome and phytohormone signaling are critical determinants of plant growth and stress resilience. This study evaluated the combined effects of *Streptomyces* sp. HU2014 and coronatine (COR) on maize (*Zea mays* L.) seedlings. Four treatments were established: control (CK), COR seed soaking (Cor), HU2014 soil inoculation (S), and combined S + Cor (SCor). Growth parameters, chlorophyll content, and antioxidant/oxidative stress markers were measured, and root and leaf transcriptomes, together with root metabolomes, were compared between SCor and CK, followed by qRT-PCR validation. Compared with CK, SCor treatment significantly increased stem diameter (~60%), plant height (~20%), and relative chlorophyll content (SPAD, ~50%). Soluble sugar levels were elevated by over 40% in both leaves and roots, accompanied by tissue-specific modulation of antioxidant enzymes. Transcriptomic analysis of SCor vs. CK revealed 2459 differentially expressed genes (DEGs) in leaves and 3444 DEGs in roots; leaves exhibited upregulation of photosynthetic pigment metabolism (porphyrin and carotenoid pathways) and volatile defense compounds (alkaloids and monoterpenoids), whereas roots showed enrichment in phenylpropanoid/flavonoid biosynthesis, benzoxazinoid synthesis, and starch/sucrose metabolism. Metabolomics of SCor vs. CK identified 526 differentially accumulated metabolites (DAMs) in roots, with significant enrichment in aminoacyl-tRNA biosynthesis, phenylalanine metabolism, and linoleic acid metabolism. Integrative multi-omics analysis further revealed that the JA precursor 13-epi-12-oxo-phytodienoic acid co-clustered with stress-responsive transcription factors (e.g., DREB1C), while tricarboxylic acid (TCA) intermediates and phenylpropanoid metabolites were linked to energy and lignin biosynthesis genes. qRT-PCR confirmed the expression trends of 14 out of 15 tested genes. Collectively, combined HU2014 and COR application triggers tissue-specific transcriptional and metabolic reprogramming in maize, coupling JA-mediated stress signaling with enhanced carbon metabolism and secondary defense compound synthesis to promote rhizosphere adaptation and seedling vigor.

## 1. Introduction

The rhizosphere serves as the core interface for intensive plant–soil–microbe interactions, regulating nutrient cycling, plant health, and ecosystem stability [1]. Plant growth and stress resistance are not isolated processes but rather outcomes of multi-interface coordination among the host, beneficial microorganisms, and the soil matrix. Plant growth-promoting rhizobacteria (PGPR) represent a key functional group within this context, promoting plant health through nutrient activation, phytohormone secretion, and pathogen suppression [2,3].

Inoculant strategies centered on beneficial microorganisms (probiotics) represent an important development direction for sustainable agriculture [4]. Prebiotics—substrates that selectively promote the proliferation of beneficial microorganisms—are likewise regarded as effective tools for regulating the rhizosphere microbiome, with root exudates, organic amendments, and specific carbon sources all capable of serving such functions [5,6]. Synbiotics, through the combination of probiotics and prebiotics, provide nutritional and competitive advantages for exogenous functional bacteria, thereby enhancing their colonization efficiency and functional stability in the rhizosphere [7,8]. However, the interaction mechanisms between the two in the rhizosphere and the tripartite combined effects among plant–microbe–substrate remain to be systematically elucidated.

The growth-promoting effects of PGPR are primarily mediated by their diverse secondary metabolites, which act as biochemical signals to shape rhizobacterial communities and regulate plant–microbe interactions [9]. The genus *Streptomyces* occupies a unique position due to its exceptional secondary metabolic capacity, producing indole compounds, siderophores, ACC deaminase, and phenazines that synergistically enhance plant growth and stress resistance [10,11]. Therefore, in addition to direct microbial inoculation, the combined application of specific plant signaling molecules with beneficial microorganisms to coordinately regulate rhizosphere interactions represents an emerging strategy for enhancing rhizosphere function. Coronatine (COR) is a polyketide phytotoxin produced by Pseudomonas syringae, functioning as a structural and functional mimic of jasmonic acid (JA). Composed of coronamic acid (CMA) and coronafacic acid (CFA), COR exhibits 100–10,000-fold greater biological activity than endogenous JA or methyl jasmonate [12]. Its molecular mechanism involves COR binding to the COI1 (coronatine insensitive 1) receptor, promoting the degradation of JAZ repressor proteins and relieving the inhibition of MYC2, thereby activating JA-responsive genes [13]. Field studies have demonstrated that COR at appropriate concentrations (e.g., 1.0 μmol L^−1^) can simultaneously increase maize yield (6.0–11.0%), water productivity (17.9%), and root development under water-deficit conditions while reducing evapotranspiration (10.0%) [14,15]. These findings establish COR as an effective molecule for modulating plant physiological status and reshaping root architecture and resource acquisition patterns.

Although COR, as a JA signaling mimic, and *Streptomyces* spp., as a biocontrol PGPR, each show broad prospects in their respective fields, their combined application in rhizosphere ecosystems has not yet been reported. Given that both converge on the JA signaling pathway—COR directly binds the COI1–JAZ co-receptor complex as a structural mimic of JA-Ile [13] whereas *Streptomyces* spp. indirectly induce systemic resistance (ISR) through JA/ET-dependent pathways [16]—their combined effects (additive, synergistic, or antagonistic) within the rhizosphere interaction network remain entirely unknown. This study systematically evaluates the individual and combined effects of COR and *Streptomyces* sp. HU2014 application on a major cereal crop. Maize was selected as the model plant due to its significant position in global agricultural production and its proven responsiveness to both COR treatment and *Streptomyces* inoculation [14,16]. To elucidate the mechanistic basis for the growth-promoting effects of combined HU2014 and COR application in maize, this study established four treatments: untreated control, COR immersion, HU2014 soil inoculation, and the combined HU2014 + COR regimen. Phenotypic traits and physiological parameters were measured. At the molecular level, the combined HU2014 + COR treatment and the control were subjected to integrated transcriptome analyses of roots and leaves, together with root metabolome profiling. Key differentially expressed genes were further validated by RT-PCR to elucidate the molecular basis underlying the combined action of the two agents from both transcriptional and metabolic perspectives. This study aimed to: (i) clarify the combined advantages of HU2014 and COR treatment on maize phenotypic and physiological indicators; (ii) systematically dissect the molecular mechanisms by which combined treatment regulates plant metabolic and stress-resistance networks based on maize root–leaf transcriptome and root metabolome data; and (iii) identify key genes and metabolic pathways responsive to the combined HU2014 + COR treatment, providing theoretical support for the combined application of plant signaling molecules and beneficial microorganisms, and facilitating green and efficient maize production.

## 2. Materials and Methods

### 2.1. Test Materials

The waxy maize cultivar Wannuo 2000 and HU2014 were provided by the Academy of Agricultural Sciences of Xinjiang Uygur Autonomous Region, in Urumqi, China. Soil samples were collected from a field in Urumqi, Xinjiang Uygur Autonomous Region (87.099344 N, 43.928544 E).

### 2.2. Determination of Physiological Indicators

After surface sterilization [17], maize seeds were randomly assigned to two soaking treatments: (i) The core reagent was a commercially available 0.006% COR formulation (Chengdu New Sunshine Crop Science Co., Ltd., Chengdu, China), applied by immersion at a 2000-fold dilution for 4 h, or (ii) immersion in sterile distilled water for 4 h. For the soil treatments, potting soil was either amended with HU2014 pellets at 4 g·kg^−1^ dry soil or left unamended. This generated four experimental combinations in a factorial arrangement: (1) COR-treated seeds sown in unamended soil (Cor); (2) COR-treated seeds sown in HU2014-amended soil (SCor); (3) water-soaked seeds sown in unamended soil (CK); and (4) water-soaked seeds sown in HU2014-amended soil (S). Each pot received three seeds, with six replicate pots per treatment (*n* = 6). Ten days after sowing, seedlings were thinned to one plant per pot. The pots were placed in a growth chamber maintained at 25 ± 2 °C with an 18/6 h light/dark cycle for four weeks. The single plant in each pot was then used as an independent biological replicate (*n* = 6 individual plants per treatment) for physiological and biochemical measurements. No pooling was performed prior to analysis. Samples from all four treatments were used to investigate the growth phenotype, measure catalase (CAT), superoxide dismutase (SOD), and malondialdehyde (MDA) activities, and soluble sugar (SS) content in maize leaves and roots according to the instructions of the respective reagent kits (Solarbio, Beijing, China) [18,19,20,21]. Total chlorophyll was monitored with the help of a relative chlorophyll content (SPAD) meter (SPAD-502, Minolta Camera Co., Ltd., Tokyo, Japan). Additional samples from the SCor and CK treatments were treated using liquid nitrogen and stored at −80 °C.

### 2.3. Transcriptome Sequencing and Validation

Total RNA was isolated from maize leaves and roots using TRIzol reagent, followed by DNase I treatment to remove genomic DNA contamination. Subsequent transcriptome sequencing and bioinformatic analyses were performed by Biomarker Technologies Co., Ltd. (Guangzhou, China). Briefly, polyadenylated mRNA was purified from total RNA with oligo(dT)-coated magnetic beads, then chemically fragmented and converted into double-stranded cDNA through sequential first-strand and second-strand synthesis. The resulting cDNA underwent end-polishing, 3′-adenylation, adapter ligation, and PCR-based enrichment to generate sequencing libraries. Quantitative real-time PCR (qRT-PCR) was performed on a QuantStudio 6 Real-Time PCR System (Applied Biosystems, Carlsbad, CA, USA) using TB Green Premix Ex Taq II (Tli RNaseH Plus) (Takara, Dalian, China). Each 20 μL reaction mixture contained 10 μL of TB Green Premix, 0.8 μL of each primer (10 μM), 2 μL of cDNA template, and 6.4 μL of RNase-free water. The thermal cycling protocol consisted of an initial denaturation at 95 °C for 30 s, followed by 40 cycles of 95 °C for 5 s and 60 °C for 30 s. A melting curve analysis was performed at the end of each run to confirm amplification specificity. Three technical replicates were used for each biological sample. Relative gene expression was calculated using the 2^−ΔΔCT^ method [22]. Paired-end 150 bp sequencing was carried out on the Illumina NovaSeq X Plus platform. Raw reads were processed with fastp (v0.18.0) for adapter trimming and quality filtering and then mapped to the maize reference genome (B73 RefGen_v4) using HISAT2 (v2.2.8). Gene-level expression was estimated as fragments per kilobase of transcript per million mapped reads (FPKM) via RNA-Seq by expectation–maximization (RSEM), and differentially expressed genes were screened using DESeq2 under the thresholds of |log_2_FC| ≥ 1 and FDR < 0.05. Functional enrichment analyses of GO terms and KEGG pathways were implemented with the clusterProfiler package.

Specific primers for genes were designed using Primer Express 5.0 software. Three candidate housekeeping genes, ubiquitin (*ZmUBQ*) and protein phosphatase 2A (*ZmPP2A*), were selected as reference genes for maize roots, whereas *ZmUBQ* and elongation factor 1-alpha (*ZmEF1-α*) were selected for maize leaves. Three replicates were used for each treatment. For each tissue, the geometric mean of the two corresponding reference genes was calculated and used for subsequent ΔCT analysis [22] (Table 1).

### 2.4. Metabolomic Analysis

Untargeted metabolomic profiling was conducted by Gene Denovo Biotechnology Co., Ltd. (Guangzhou, China). Samples of maize roots were pulverized in liquid nitrogen and metabolites were extracted using a methanol–water mixture (4:1, *v*/*v*), followed by vortexing, sonication, centrifugation, and filtration through 0.22 μm membranes. Metabolic profiling was performed using an ultra-high-performance liquid chromatography–tandem mass spectrometry (UHPLC-MS/MS) system consisting of a Waters ACQUITY UPLC coupled to a Thermo Q Exactive HF-X mass spectrometer (Thermo Fisher Scientific Inc., Bremen, Germany), equipped with an ACQUITY UPLC BEH C18 column (1.7 μm, 2.1 × 100 mm), operating in both positive and negative ion modes to maximize metabolite coverage. Quality control (QC) samples were regularly interspersed to monitor system stability and data reproducibility. Raw data were processed using Compound Discoverer 3.1 for peak extraction, alignment, and annotation against the mzCloud, KEGG, HMDB, and in-house spectral libraries, with metabolites classified into Level 1 (standard confirmation), Level 2 (MS/MS match), and Level 3 (predicted) based on confidence levels. Multivariate statistical analyses, including principal component analysis (PCA) and orthogonal partial least squares discriminant analysis (OPLS-DA), were performed using SIMCA software (version 17.0) and R packages (version 1.42.0). Differential metabolites were screened based on variable importance in projection (VIP) ≥ 1 and Student’s *t*-test *p* < 0.05, followed by KEGG pathway enrichment, MSEA (Metabolite Set Enrichment Analysis), and topological analysis to elucidate their biological functions.

### 2.5. Statistical Analysis

All values are presented as the mean ± standard deviation (SD) of three independent biological replicates. One-way analysis of variance (ANOVA), followed by Duncan’s multiple range test, was employed to assess significant differences (*p* < 0.05) using SPSS software (version 16.0; SPSS Inc., Chicago, IL, USA). Additionally, simple linear regression analysis was performed using the same statistical package.

## 3. Results

### 3.1. Effects of HU2014 Treatment on Maize Seedling Growth

**Figure 1:**
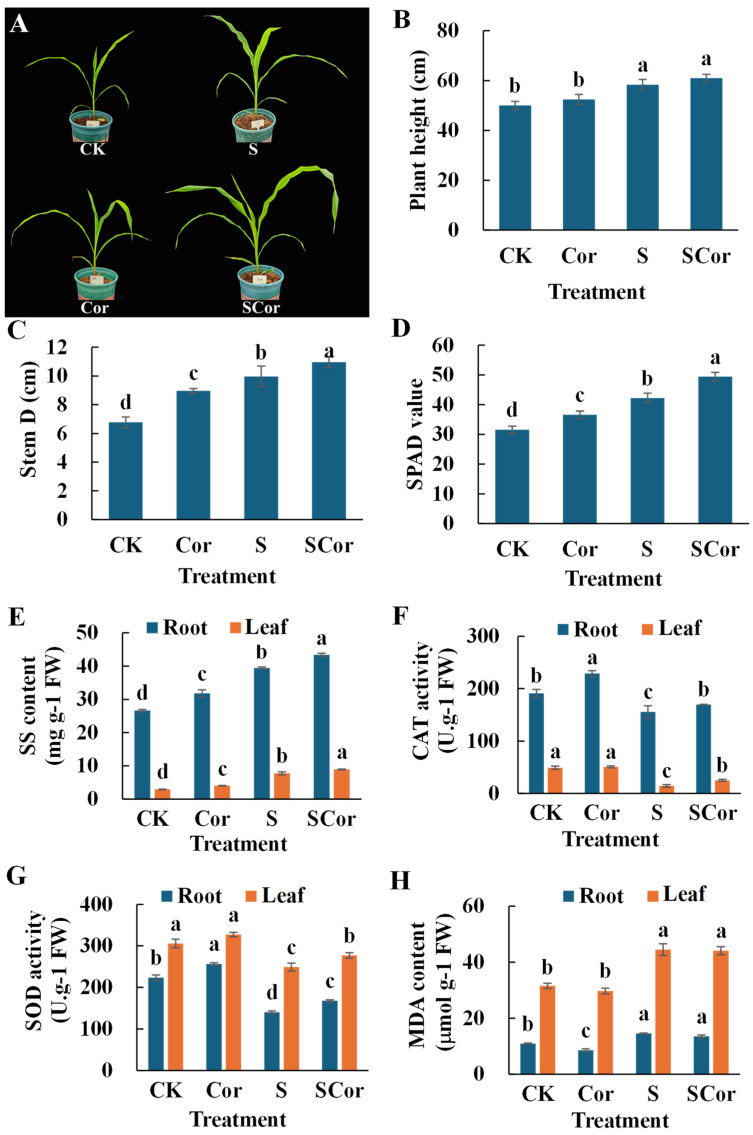
Effects of HU2014 and coronatine, alone or in combination, on maize phenotype and physiological–biochemical indices. (**A**) Representative leaf phenotype; (**B**) Plant height; (**C**) Stem diameter; (**D**) Leaf relative chlorophyll content (SPAD); (**E**) Soluble sugar content in leaves and roots; (**F**) Catalase (CAT) activity; (**G**) Superoxide dismutase (SOD) activity; (**H**) Malondialdehyde (MDA) content. D, diameter; FW, fresh weight. Values are means ± SD of six independent biological replicates. Bars with different letters are significantly different at *p* < 0.05 (Duncan’s test).

This experiment examined the phenotypic, physiological, and biochemical indices of maize seedlings subjected to four treatments (Figure 1). Growth parameters revealed differential treatment effects. Plant height in the S and SCor groups was significantly greater than that in the CK and Cor groups (*p* < 0.05), whereas no significant difference was detected between CK and Cor or between S and SCor (Figure 1A,B). In contrast, stem diameter and leaf SPAD values increased progressively and significantly across all four treatments in the order CK < Cor < S < SCor, with each pairwise comparison reaching statistical significance (*p* < 0.05) (Figure 1C,D). SS content in both roots and leaves followed a consistent gradient of CK < Cor < S < SCor, with significant differences separating all treatments (*p* < 0.05) (Figure 1E).

Antioxidant enzyme activities displayed complex, tissue-specific patterns (Figure 1F–H). CAT activity in roots was highest in the Cor treatment when compared to CK (*p* < 0.05), intermediate in the SCor treatment (*p* > 0.05), and lowest in the S treatment (*p* < 0.05). In leaves, CAT activity was not significantly changed in the Cor group (*p* > 0.05), significantly reduced in both the S and SCor groups (*p* < 0.05) (Figure 1F). For SOD activity, roots exhibited the highest levels in the Cor groups (*p* < 0.05), intermediate levels in the SCor group (*p* < 0.05), and the lowest activity in the S group (*p* < 0.05). In leaves, SOD activity was highest in the Cor group (*p* < 0.05), followed by the SCor group (*p* < 0.05), and lowest in the S group (*p* < 0.05) (Figure 1G). Lipid peroxidation, as reflected by MDA content, showed a consistent pattern in both roots and leaves: the S and SCor groups displayed significantly elevated MDA levels (*p* < 0.05), whereas the Cor treatment actually reduced MDA content below that of CK (Figure 1H).

### 3.2. Transcriptomic Profiling of Maize Roots and Leaves in Response to HU2014

**Figure 2:**
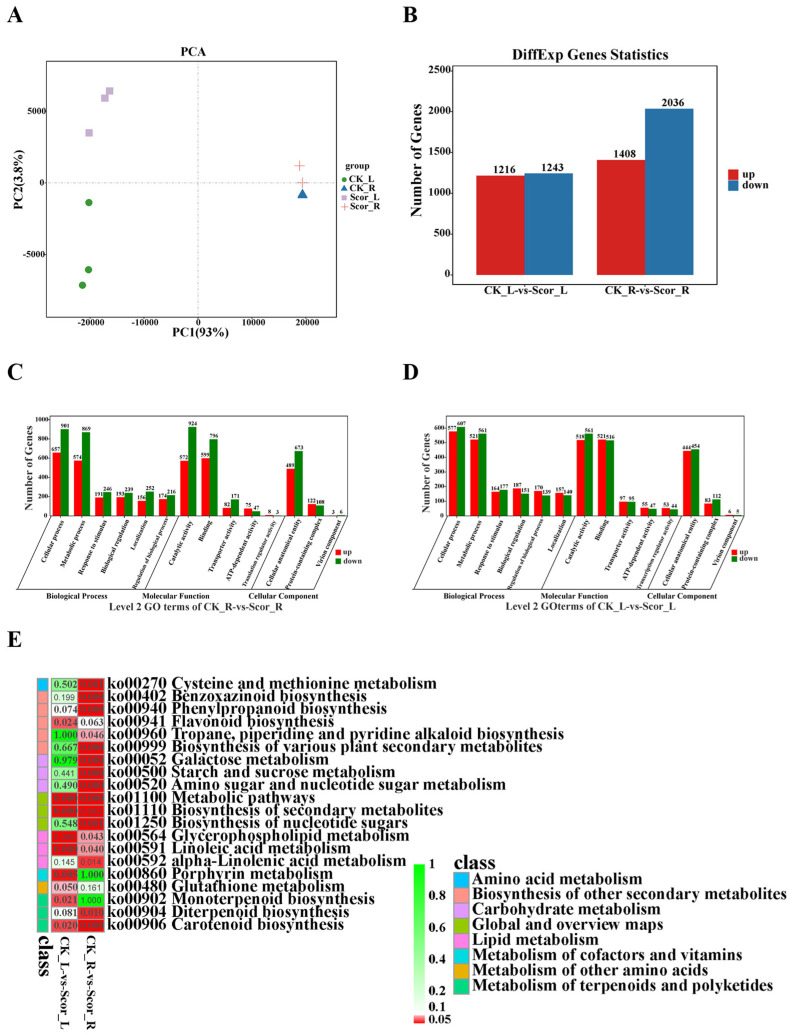
Transcriptomic analyses of maize leaves and roots in response to SCor treatment. (**A**) Principal component analysis (PCA) showing tissue-specific clustering of transcriptomes. (**B**) Statistics of differentially expressed genes (DEGs) between CK and SCor in leaves and roots. (**C**,**D**) Gene Ontology (GO) enrichment analysis of DEGs in leaves (**C**) and roots (**D**), respectively. (**E**) Top 20 enriched KEGG pathways in leaves and roots. CK_L, SCor_L, CK_R and SCor_R represent control maize leaves, combined treatment (HU2014 + COR) maize leaves, control maize roots and combined treatment (HU2014 + COR) maize roots, respectively.

We performed transcriptomic analyses of both leaves and roots to uncover the gene regulatory mechanisms underlying the beneficial effects of SCor treatment on maize. According to the PCA (Figure 2A), the transcriptomes exhibited distinct tissue-specific clustering, with leaf samples clearly separated from root samples along PC1, and treatment groups showed separation from their respective controls within each tissue. A total of 2459 differentially expressed genes (DEGs) were identified in leaves, including 1216 upregulated and 1243 downregulated genes in the combined SCor treatment compared to the control. In roots, 3444 DEGs were identified, with 1408 upregulated and 2036 downregulated genes under the same treatment (Figure 2B). Gene Ontology (GO) enrichment analyses revealed that DEGs in both tissues were enriched across Biological Process (cellular process, metabolic process, response to stimulus), Molecular Function (catalytic activity, binding, transporter activity), and Cellular Component categories (Figure 2C,D). KEGG pathway enrichment analysis revealed significant tissue-specific differences in metabolic reprogramming between maize roots and leaves (Figure 2E). In leaves, the top five KEGG pathways (ranked by enrichment significance) comprised tropane, piperidine and pyridine alkaloid biosynthesis, porphyrin metabolism, carotenoid biosynthesis, biosynthesis of various plant secondary metabolites, and monoterpenoid biosynthesis, indicating predominant activation of photosynthetic pigment metabolism (porphyrin and carotenoid pathways) and volatile defense compound synthesis. Conversely, root tissues exhibited enrichment in flavonoid biosynthesis, phenylpropanoid biosynthesis, benzoxazinoid biosynthesis, cysteine and methionine metabolism, and starch and sucrose metabolism, reflecting enhanced chemical defense and energy allocation processes. Additionally, it is noteworthy that both roots and leaves showed significant enrichment in metabolic pathways and biosynthesis of secondary metabolites, indicating functional convergence at the overall metabolic network level between the two tissues, yet with distinct metabolic branch specialization–leaves focusing on photosynthetic pigments and volatile defense substances, whereas roots emphasized phenolic defense compounds and energy metabolism regulation.

### 3.3. Metabolic Responses of Maize Roots to HU2014 Inoculation

**Figure 3:**
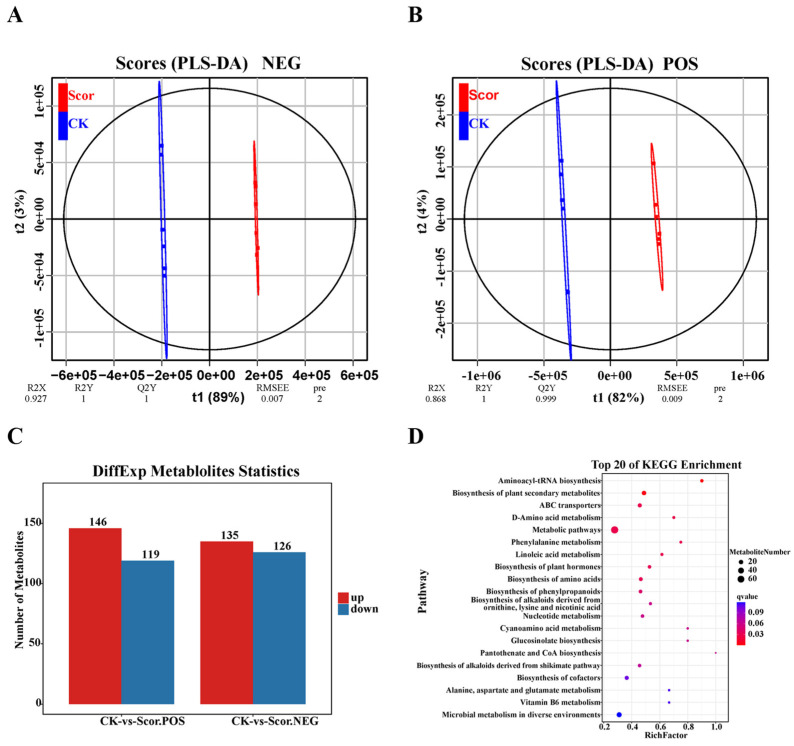
Metabolomic analysis was performed on maize roots from CK and SCor treatments. (**A**,**B**) Partial least squares discriminant analysis (PLS-DA) score plots on negative (NEG, (**A**)) and positive (POS, (**B**)) ion modes. (**C**) DAM statistics showing the number of up- and down-regulated metabolites in positive (POS) and negative (NEG) ion modes. (**D**) Top 20 enriched KEGG pathways of differential metabolites. CK and SCor represent control and combined-treatment (HU2014 + COR) maize roots, respectively.

Metabolomic analysis revealed that SCor treatment significantly altered the metabolite composition in maize roots. According to the PLS-DA score plots (Figure 3A,B), distinct separation was observed between the treatment (SCor) and control (CK) groups under both positive (POS) and negative (NEG) ionization modes (POS: R^2^Y = 1, Q^2^Y = 0.999; NEG: R^2^Y = 1, Q^2^Y = 1), indicating robust predictive capability and reproducibility of the models, as well as significant metabolic differences between the two groups. A total of 526 differentially accumulated metabolites (DAMs) were identified, comprising 146 up-regulated and 119 down-regulated metabolites in the positive ion mode and 135 up-regulated and 126 down-regulated metabolites in the negative ion mode (Figure 3C). KEGG pathway enrichment analysis (Figure 3D) demonstrated that these DAMs were predominantly enriched in aminoacyl-tRNA biosynthesis, the biosynthesis of various plant secondary metabolites, metabolic pathways, phenylalanine metabolism, and linoleic acid metabolism. These findings indicate that SCor treatment substantially activated amino acid metabolism, defensive secondary metabolite synthesis, and lipid signaling processes in roots, reflecting coordinated carbon–nitrogen metabolic reallocation and the accumulation of defense-related chemicals during rhizosphere microbe–plant interactions.

### 3.4. Integration of Transcriptomic and Metabolomic Data

**Figure 4:**
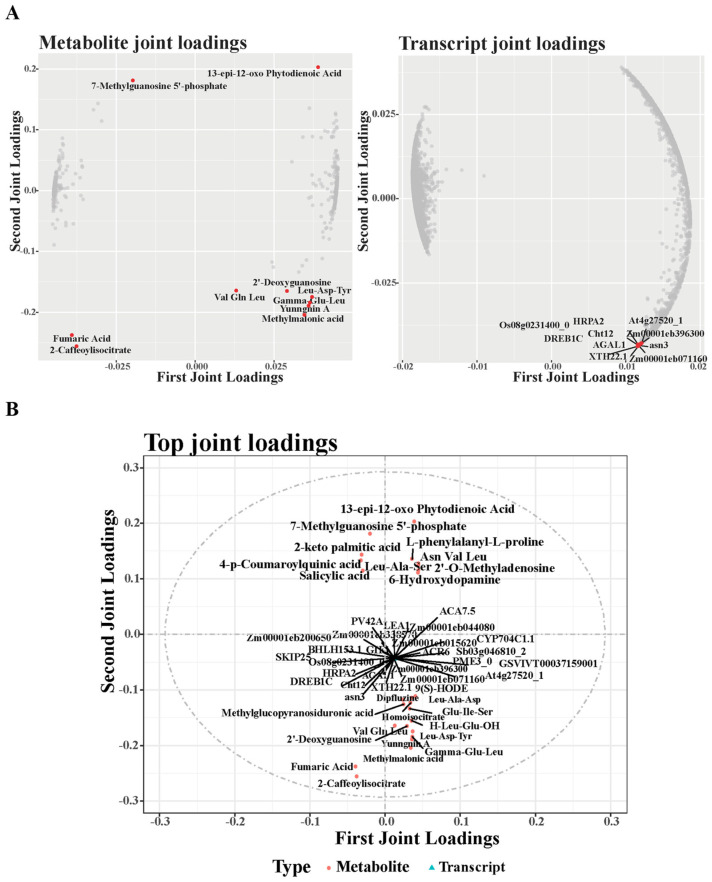
Combined transcriptomic and metabolomic data uncovered systems-level alterations in maize roots under SCor treatment. (**A**) Metabolite joint loadings plot and transcript joint loadings plot. (**B**) Top joint loadings biplot integrating both metabolites (pink dot marker) and transcripts (blue triangular marker).

To elucidate the coordinated regulation between gene expression and metabolic profiles, we performed an integrated multi-omics analysis of transcriptomic and metabolomic data from maize roots following SCor treatment (Figure 4A,B). The joint loadings plot revealed strong correlations between specific DEGs and DAMs. Notably, the oxylipin pathway metabolite 13-epi-12-oxo phytodienoic acid (OPDA) clustered closely with stress-responsive transcripts including dehydration-responsive element-binding protein 1C (DREB1C), HRPA2, and AGAL1, suggesting that HU2014-induced jasmonate signaling may coordinate with drought/stress-responsive transcription factors to enhance root resilience. Additionally, purine-related metabolites such as 7-Methylguanosine 5′-phosphate and 2′-deoxyguanosine showed tight associations with cell cycle and DNA repair genes, reflecting enhanced cellular activity upon bacterial colonization. The tricarboxylic acid (TCA) cycle intermediate Fumaric Acid and phenylpropanoid-related 2-caffeoylisocitrate were linked to genes involved in energy metabolism and lignin biosynthesis, respectively. These multi-omics associations demonstrate that SCor treatment triggers a systems-level response in maize roots, wherein hormonal signaling (particularly oxylipin pathways), energy metabolism, and stress adaptation are transcriptionally and metabolically coupled to support rhizosphere establishment and plant growth promotion.

### 3.5. Validation by Rt-Pcr

**Figure 5:**
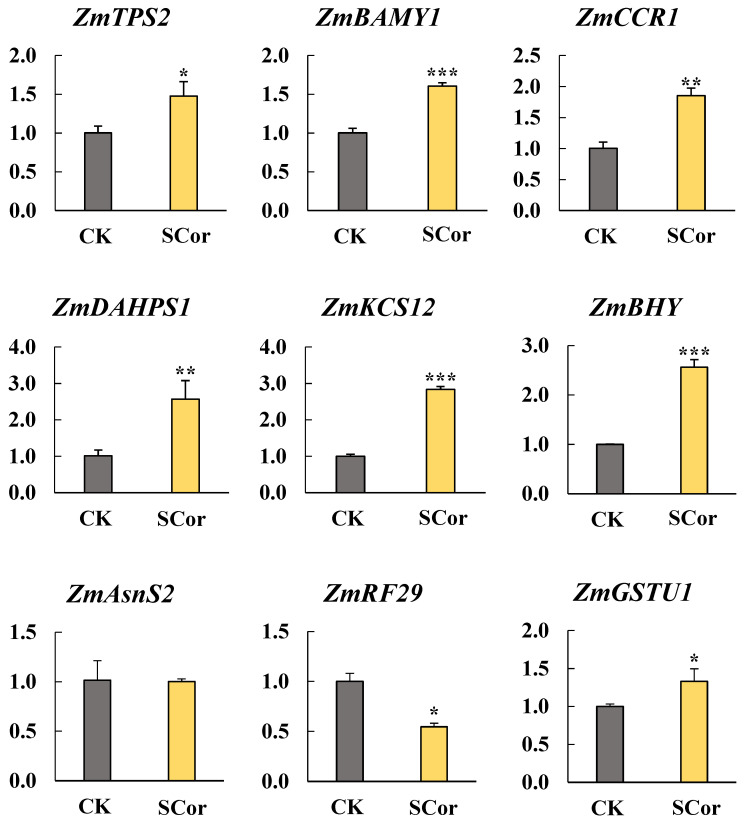
Validation of RNA-seq data by qRT-PCR. Relative expression levels of nine differentially expressed candidate genes (*ZmTPS2*, *ZmBAMY1*, *ZmCCR1*, *ZmDAHPS1*, *ZmKCS12*, *ZmBHY*, *ZmAsnS2*, *ZmRF29*, and *ZmGSTU1*) in CK and SCor groups were determined by qRT-PCR. Data are presented as mean ± S.D. (*n* = 3). Significant differences between the CK and SCor groups were assessed by an unpaired *t* test and indicated by asterisks (ns means no significant, * *p* < 0.05, ** *p* < 0.01, *** *p* < 0.001).

**Figure 6:**
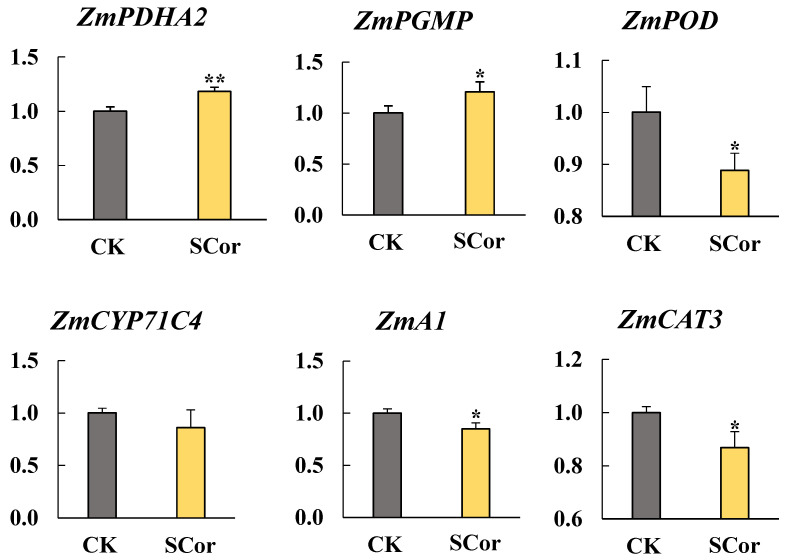
qRT-PCR validation of additional differentially expressed genes. Relative expression levels of six candidate genes (*ZmPDHA2*, *ZmPGMP*, *ZmPOD*, *ZmCYP71C4*, *ZmA1*, and *ZmCAT3*) in the control (CK) and treatment (SCor) groups were determined by qRT-PCR. Data are presented as mean ± S.D. (*n* = 3). Significant differences between CK and SCor groups were assessed by an unpaired *t* test and indicated by asterisks (ns means no significant, * means *p* < 0.05, ** means *p* < 0.01).

To validate the RNA-seq data, nine DEGs (Table 2) from maize leaves were selected for qRT-PCR verification. As shown in Figure 5, the expression trends of eight genes were consistent with the transcriptomic data. Up-regulated genes included Z*mTPS2*, *ZmBAMY1*, *ZmCCR1*, *ZmDAHPS1*, *ZmKCS12*, *ZmBHY*, and Z*mGSTU1*; the down-regulated gene *ZmRF29* is involved in auxin signaling. The qRT-PCR result for *ZmAsnS2* showed no significant difference between the control and SCor treatments, diverging from the transcriptomic data and requiring further verification. Six DEGs (Table 2) from maize roots were also validated (Figure 6). The expression trends of two up-regulated genes, *ZmPDHA2* and *ZmPGMP*, and four down-regulated genes, *ZmPOD*, *ZmCYP71C4*, *ZmA1*, and *ZmCAT3*, were all consistent with the transcriptomic data. Up-regulated genes were mainly involved in carbon metabolism (pyruvate dehydrogenase and phosphoglucomutase), whereas down-regulated genes covered antioxidant enzymes (*ZmPOD* and *ZmCAT3)*, secondary metabolism (*ZmCYP71C4* and *ZmA1*), with ZmA1 specifically functioning in flavonoid/anthocyanin biosynthesis.

Absolute fold changes may vary between RNA-seq and qRT-PCR due to technical differences; however, the directional consistency supports the reliability of transcriptomic data.

## 4. Discussion

This study evaluated the individual and combined growth-promoting effects of HU2014 and coronatine (COR) on maize seedlings. The combined treatment (SCor) significantly enhanced specific growth traits, including stem diameter, chlorophyll content, and soluble sugar accumulation, compared to the individual treatments, suggesting a composite action of the bacterial inoculant and the JA mimic. Transcriptomic and metabolomic analyses further revealed tissue-specific metabolic reprogramming associated with the combined treatment: leaves exhibited enhanced photosynthetic pigment metabolism and volatile defense compound synthesis, whereas roots displayed activation of phenylpropanoid/flavonoid and benzoxazinoid biosynthesis alongside energy metabolism.

### 4.1. Growth Promotion and Redox Homeostasis

The SCor treatment substantially enhanced maize vegetative growth, achieving the greatest stem diameter, plant height, and chlorophyll content among all treatments, while also attaining the highest SS accumulation in both roots and leaves. Notably, root SS concentrations were substantially higher than leaf SS concentrations across all groups, suggesting preferential carbon allocation to below-ground tissues under microbial influence. Phenotypically, COR alone significantly promoted stem thickening and chlorophyll accumulation and reduced MDA levels, indicating membrane protection, which is consistent with previous reports [15,23,24]. HU2014 alone also significantly promoted plant height, SS accumulation, and stem thickness, yet suppressed both CAT and SOD activities while elevating MDA levels. The combined SCor treatment integrated these advantages, obtaining the most robust vegetative phenotype and the highest SS content, while simultaneously modulating antioxidant enzyme activities and MDA levels in maize roots and leaves.

Notably, plant height did not differ significantly between the S and SCor groups (Figure 1B), whereas stem diameter, SPAD value, and soluble sugar content showed progressive increases across CK < Cor < S < SCor. This trait-specific pattern suggests that the combined treatment does not uniformly enhance all growth parameters; rather, it preferentially promotes carbon allocation to structural thickening (stem diameter), photosynthetic capacity (chlorophyll), and osmolyte accumulation (soluble sugars) rather than vertical elongation. This growth promotion was accompanied by elevated MDA levels and tissue-specific modulation—rather than simple upregulation—of antioxidant enzyme activities. The decrease in CAT and SOD activities alongside increased soluble sugars and elevated MDA suggests that HU2014 may induce a controlled oxidative priming response rather than severe oxidative damage. In plant–microbe interactions, transient reactive oxygen species (ROS) accumulation often serves as a signaling cue that triggers systemic resistance and metabolic reprogramming [25,26,27,28]. However, we acknowledge that the elevated MDA in the S and SCor groups may alternatively represent a physiological ‘cost’ of accelerated growth and enhanced carbon metabolism, rather than purely a stress signal or an indicator of membrane damage. MDA is a downstream product of lipid peroxidation, but its accumulation can also reflect high metabolic turnover rates in rapidly expanding tissues. The concomitant rise in soluble sugars may function both as osmolytes and as carbon skeletons for secondary metabolite biosynthesis, fueling the downstream defense and growth responses observed at the transcriptomic level [29,30,31]. Thus, SCor does not simply mirror the S treatment but rather integrates the growth-promoting attributes of both agents, with the sustained oxidative profile potentially reflecting a metabolic cost of accelerated rhizosphere colonization and biomass accumulation rather than a detrimental stress response.

### 4.2. Transcriptomic Reprogramming

Transcriptomic analysis revealed profound tissue-specific reprogramming in response to the combined SCor treatment. In leaves, the upregulation of porphyrin and carotenoid metabolism aligned with the increased SPAD values, suggesting enhanced stability of the photosynthetic apparatus. The concomitant activation of monoterpenoid and alkaloid biosynthesis indicated that the combined treatment primed above-ground tissues for volatile-mediated defense and photoprotection. This transcriptional pattern was corroborated by qRT-PCR validation, which confirmed the upregulation of *ZmTPS2* and *ZmBHY*—enzymes that channel carbon into terpenoid and carotenoid pathways, respectively—supporting enhanced terpenoid and carotenoid metabolism for photoprotection and volatile defense. In roots, the enrichment of phenylpropanoid, flavonoid, and benzoxazinoid pathways pointed to intensified chemical defense in the rhizosphere. Phenylpropanoids and benzoxazinoids are hallmark defensive metabolites in grasses that deter soil-borne pathogens and mediate allelopathic interactions [32,33,34,35]. The upregulation of *ZmCCR1* and *ZmDAHPS1*, as validated by qRT-PCR, supported a metabolic flux toward phenylpropanoid-derived defense compounds and aromatic amino acid biosynthesis. Notably, the downregulation of antioxidant genes *ZmPOD* and *ZmCAT3* in roots, despite elevated MDA, aligns with our ‘controlled oxidative priming’ hypothesis, wherein reduced antioxidant enzyme activity may reflect a signaling state rather than oxidative damage. Furthermore, the downregulation of *ZmRF29* suggests that the combined treatment modulates auxin signaling, potentially redirecting growth resources from vertical elongation to structural thickening, consistent with the observed increase in stem diameter but not in plant height.

Another notable observation is that the qRT-PCR result for *ZmAsnS2* showed no significant difference between the control and the SCor treatment, diverging from the transcriptomic data. This discrepancy may be attributed to several factors, including (i) post-transcriptional regulation, such as microRNA-mediated mRNA degradation or translational repression, which can decouple transcript abundance from protein levels; (ii) differences in primer amplification efficiency or splice-variant coverage between qRT-PCR and RNA-seq; and (iii) tissue heterogeneity or temporal expression dynamics that may not be fully captured by the two methods. Further investigation, including protein-level validation, is warranted to resolve this discrepancy.

### 4.3. Metabolomic Remodeling in Maize Roots

Untargeted metabolomic profiling of roots further resolved the metabolic consequences of this transcriptional shift. A total of 526 differentially accumulated metabolites (DAMs) were identified, with distinct metabolic signatures separating SCor-treated samples from controls under both positive and negative ion modes. KEGG enrichment analysis revealed that DAMs were significantly enriched in phenylalanine metabolism and linoleic acid metabolism, providing a mechanistic basis for the observed flux into phenylpropanoid and oxylipin pathways. The metabolic activation of linoleic acid likely fed into the accumulation of OPDA, a key precursor of jasmonic acid (JA) biosynthesis. Additionally, the TCA cycle intermediate fumaric acid was significantly altered, suggesting enhanced carbon flux into energy-producing pathways to support the metabolic demands of microbial colonization.

### 4.4. Multi-Omics Integration: JA Signaling and Carbon–Energy Coupling

Integrative analysis of transcriptomic and metabolomic datasets revealed that the JA signaling axis serves as a central mediator of the HU2014–COR interaction. OPDA co-clustered with stress-responsive transcripts, including DREB1C in the joint loadings plot, indicating transcriptional–metabolic coupling of the oxylipin pathway with drought/stress adaptation networks. COR functions as a structural mimic of JA-Ile, directly binding the COI1–JAZ co-receptor complex to derepress MYC2 and activate JA-responsive genes [36,37,38]. Meanwhile, *Streptomyces* spp. are known to induce systemic resistance via JA/ethylene-dependent pathways [39,40,41]. Our data suggest that the combined application amplifies this endogenous signal, translating into enhanced transcription of stress-adaptive and defense-related genes. This hormonal signaling is further coupled with carbon–energy metabolism. The upregulation of *ZmPDHA2* and *ZmPGMP* indicates enhanced carbon flux from glycolysis into the TCA cycle and subsequent biosynthetic pathways, likely providing the ATP and reductant necessary for sustaining growth and defense simultaneously. Collectively, these multi-omics associations demonstrate that SCor treatment triggers a systems-level response in maize roots, wherein JA-mediated signaling, energy metabolism, and stress adaptation are transcriptionally and metabolically coupled to support plant growth promotion.

It should be noted that the transcriptomic and metabolomic analyses were performed only between SCor and CK. Consequently, the identified DEGs and DAMs reflect the overall molecular response to the combined treatment and cannot be attributed exclusively to a synergistic interaction between COR and HU2014. Future studies incorporating single-treatment omics profiles are required to disentangle synergistic, additive, and independent regulatory effects.

In conclusion, our findings support a model which combined COR and HU2014 application remodels maize metabolism through a JA-centered signaling network. Above ground, this manifests as enhanced photosynthetic pigment metabolism and volatile defense priming; below ground, it activates phenylpropanoid–benzoxazinoid chemical defense and reconfigures carbon–energy metabolism to support rhizosphere colonization and root growth. These results provide a theoretical foundation for the combined application of JA mimics and actinobacterial inoculants in maize production.

## 5. Conclusions

This study evaluated the individual and combined growth-promoting effects of COR and HU2014 on maize seedlings. The SCor treatment promoted maize seedling growth, outperforming single treatments in biomass, chlorophyll, and soluble sugar accumulation. Mechanistically, this involved tissue-specific metabolic reprogramming: leaves enhanced photosynthetic pigment and volatile defense compound synthesis, while roots activated phenylpropanoid/flavonoid and benzoxazinoid biosynthesis alongside energy metabolism. Multi-omics integration further revealed that COR and HU2014 converge on the jasmonate signaling axis, wherein the JA precursor OPDA coordinated with stress-responsive transcription factors (e.g., *DREB1C*) and TCA/phenylpropanoid metabolites to couple stress adaptation with carbon metabolism, thereby driving rhizosphere colonization and seedling vigor. The study provides a theoretical basis for the combined application of plant signaling molecules and beneficial actinobacteria. Nevertheless, the combined HU2014–COR strategy, through microbiome–phytohormone co-regulation, demonstrates potential for green and efficient maize production.

## Figures and Tables

**Table 1:** Quantitative PCR primer sequences used in this study.

Gene Name	Forward and Reverse Primer (5′-3′)
pyruvate dehydrogenase E1 component subunit alpha-2 (*ZmPDHA2*)	GGTCTGCGAGAACAAC
	AGCCAGGCACATAGTC
phosphoglucomutase (*ZmPGMP*)	AACTCTAGCCGACCCAT
	GAAAGCACAAGGCAAAG
peroxidase 2 (*ZmPOD*)	CTCCTCCGCCTCCATTT
	AAGCCCGTCAGTTCAGTC
indole-2-monooxygenase (*ZmCYP71C4*)	GCGATGCTGTCCAACC
	CGAGACCGTGATCCCAAA
dihydroflavonol 4-reductase (*ZmA1*)	AGGCGATAATGGAGGGA
	CGGAGCGAATCAGAGTTT
catalase isozyme 3 (*ZmCAT3*)	ACACCCAAAGGTCAGCC
	TGCAAAGCAATCGTCGTAT
terpene synthase 2, chloroplastic-like (*ZmTPS2*)	GCTGGCTGTTGAGAAG
	AGCAGGAGCAAGAATG
beta-amylase (*ZmBAMY1*)	GGAGGGAAAGCAGGGAG
	GCACCGAATCAGGCAGT
cinnamoyl-CoA reductase 1-like (*ZmCCR1*)	CCCTCCGCAAGTCCTTC
	CTGCTTCCTCGGGTTCA
phospho-2-dehydro-3-deoxyheptonate aldolase 1 (*ZmDAHPS1*)	ATTGGCACATCGGGTTG
	GCTCCATCTTGTCGCTCAC
3-ketoacyl-CoA synthase 12 (*ZmKCS12*)	GGGGAGCGGCTTCGTAT
	TGATGAAACTTGGTCGGATT
beta-carotene hydroxylase (*ZmBHY*)	GACGCCACCCAATAAGAC
	GAGACCCTCGCAACCTG
asparagine synthetase 2 (*ZmAsnS2*)	AGAAGAGTTCGTGGACAT
	GCACCCAGATTGTTATG
auxin response factor 29 (*ZmRF29*)	TCCACCTTCCTATTACC
	ACTGCCGATGAACTCT
glutathione transferase 6 (*ZmGSTU1*)	CTGGACTTCTGGGTGA
	AGGATGACGAGCGACT
Ubiquitin	CACCTTGTGCCAATACA
	TCTGACCATCCAACCTC
elongation factor 1-alpha (*ZmEF1-*α)	TATGGTGAAGATGGTTCCCACTAAG
	TTGGCTCCAGTTGGGTCCTT
protein phosphatase 2A (*ZmPP2A*)	CTTCAGCATTTCGGTCGT
	TTGCCTCTTTATCTCCTTCTCC

**Table 2:** Functional annotations of selected differentially expressed genes (DEGs) in leaves and roots.

Organism	Gene	Function
Leaf	*ZmTPS2*	Encodes a terpene synthase that channels carbon into monoterpenoid biosynthesis, contributing to volatile defense compound production.
	*ZmBHY*	Encodes a beta-carotene hydroxylase involved in carotenoid biosynthesis and photoprotection.
	*ZmBAMY1*	Encodes a beta-amylase that participates in starch degradation and soluble sugar mobilization.
	*ZmCCR1*	Encodes a cinnamoyl-CoA reductase, a key enzyme in lignin biosynthesis via the phenylpropanoid pathway.
	*ZmDAHPS1*	Encodes a phospho-2-dehydro-3-deoxyheptonate aldolase, catalyzing the first committed step in the shikimate pathway for aromatic amino acid and secondary metabolite synthesis.
	*ZmKCS12*	Encodes a 3-ketoacyl-CoA synthase involved in very-long-chain fatty acid and cuticular wax biosynthesis.
	*ZmGSTU1*	Encodes a glutathione S-transferase functioning in detoxification and redox homeostasis.
	*ZmRF29*	Encodes an auxin response factor, and its downregulation suggests attenuated auxin signaling.
	*ZmAsnS2*	Encodes an asparagine synthetase involved in nitrogen assimilation and amino acid metabolism.
Root	*ZmPDHA2*	Encodes the E1 alpha subunit of pyruvate dehydrogenase, linking glycolysis to the TCA cycle.
	*ZmPGMP*	Encodes phosphoglucomutase, catalyzing the interconversion of glucose-1-phosphate and glucose-6-phosphate in glycolysis and starch metabolism.
	*ZmPOD*	Encodes peroxidase, a central antioxidant enzyme.
	*ZmCAT3*	Encodes catalase, a central antioxidant enzyme.
	*ZmCYP71C4*	Encodes a cytochrome P450 monooxygenase involved in benzoxazinoid biosynthesis.
	*ZmA1*	Encodes dihydroflavonol 4-reductase, a key enzyme in flavonoid/anthocyanin biosynthesis.

## Data Availability

The original contributions presented in this study are included in the article. Further inquiries can be directed to the corresponding authors.
